# Disrupted neural activity patterns to novelty and effort in young adult *APOE*‐e4 carriers performing a subsequent memory task

**DOI:** 10.1002/brb3.612

**Published:** 2017-01-05

**Authors:** Simon Evans, Nicholas G. Dowell, Naji Tabet, Sarah L. King, Samuel B. Hutton, Jennifer M. Rusted

**Affiliations:** ^1^ School of PsychologyUniversity of SussexBrightonEast SussexUK; ^2^ School of PsychologyUniversity of SurreyGuildfordSurreyUK; ^3^ Brighton and Sussex Medical School (BSMS)BrightonEast SussexUK

**Keywords:** *APOE*, memory, fMRI, reflex, pupillary, hippocampus

## Abstract

**Introduction:**

The APOE e4 allele has been linked to poorer cognitive aging and enhanced dementia risk. Previous imaging studies have used subsequent memory paradigms to probe hippocampal function in e4 carriers across the age range, and evidence suggests a pattern of hippocampal overactivation in young adult e4 carriers.

**Methods:**

In this study, we employed a word‐based subsequent memory task under fMRI; pupillometry data were also acquired as an index of cognitive effort. Participants (26 non‐e4 carriers and 28 e4 carriers) performed an incidental encoding task (presented as word categorization), followed by a surprise old/new recognition task after a 40 minute delay.

**Results:**

In e4 carriers only, subsequently remembered words were linked to increased hippocampal activity. Across all participants, increased pupil diameter differentiated subsequently remembered from forgotten words, and neural activity covaried with pupil diameter in cuneus and precuneus. These effects were weaker in e4 carriers, and e4 carriers did not show greater pupil diameter to remembered words. In the recognition phase, genotype status also modulated hippocampal activity: here, however, e4 carriers failed to show the conventional pattern of greater hippocampal activity to novel words.

**Conclusions:**

Overall, neural activity changes were unstable in e4 carriers, failed to respond to novelty, and did not link strongly to cognitive effort, as indexed by pupil diameter. This provides further evidence of abnormal hippocampal recruitment in young adult e4 carriers, manifesting as both up and downregulation of neural activity, in the absence of behavioral performance differences.

## 1 Introduction

In humans, three variants of the *APOE* gene exist (e2, e3, e4). The e4 allelic variant has been the focus of considerable recent research activity due to it being a well‐established risk factor for Alzheimer's disease (AD) (Rocchi, Pellegrini, Siciliano, & Murri, 2003). It also impacts healthy aging: carriers of the e4 variant (from this point referred to as e4+) have been shown (in the absence of AD) to be cognitively disadvantaged in later life relative to non‐e4 carriers (e4−) on measures of episodic memory, executive functioning and overall global cognitive ability (Wisdom, Callahan, & Hawkins, 2011), and longitudinal studies suggest that healthy age‐related cognitive decline begins earlier in e4+ and progresses quicker (Caselli et al., 2009; Davies et al., 2012). These effects occur in the context of brain structural differences. Healthy older e4+ show gray matter (GM) reductions in hippocampal and frontotemporal regions (Wishart et al., 2006); this is noteworthy since these regions are among the first to atrophy in AD (Thompson et al., 2003). Neural activation differences are also evident, with greater BOLD activity observed in various regions including precuneus, frontal, and right hippocampal regions during picture encoding in healthy e4+ aged 70–80 (Bondi, Houston, Eyler, & Brown, 2005). Retrieval of memorized word pairs has also been shown to induce greater activity in parietal, and prefrontal and hippocampal regions in e4+ (aged 47–82), with degree of overactivity correlating with degree of memory decline measured 2 years later (Bookheimer et al., 2000). Overactivity has also been demonstrated during working memory tasks, with e4+ aged 50–75 showing greater recruitment of medial frontal and parahippocampal areas (Filbey, Chen, Sunderland, & Cohen, 2010). Another study reported increased activity in prefrontal, temporal and parietal regions during memory encoding, but coupled with frontal decreases during retrieval, in e4+ aged 55–65 (Kukolja, Thiel, Eggermann, Zerres, & Fink, 2010). These findings have been interpreted as representing compensatory mechanisms: e4+ recruit additional neural resources to maintain cognitive performance (Tuminello & Han, 2011), thus requiring additional cognitive effort to achieve comparable performance levels to their none4 peers (Bondi et al., 2005).

There is some evidence that e4+ might show neural differences even in young adulthood. Most work has focused on hippocampal activity patterns to try and characterize differences that might anticipate later‐life pathology, and various studies point to a pattern of hippocampal overactivity in e4+. Dennis et al. (2009) employed a subsequent memory task: this paradigm begins with an acquisition phase containing a set of stimuli to be remembered, followed after some fixed interval by a recognition phase where those same stimuli are presented again, interleaved with some novel stimuli, and participants respond to indicate whether they think each item was previously studied or novel. Dennis et al. employed pictorial stimuli and a 24‐hour retention period, and investigated activation in the medial temporal lobe during the acquisition phase, comparing activity to items that were subsequently remembered and items that were subsequently forgotten. In adults aged 20–25, hippocampal activity in e4− did not differentiate remembered from forgotten, but significantly greater bilateral hippocampal recruitment to subsequently remembered items was seen in e4+. Task performance was equal across genotypes. Similarly, a study by Filippini et al. (2009) used a variant of the subsequent memory paradigm, again using pictorial stimuli but focusing on the recognition phase, comparing effects of novel versus familiar stimuli. It was found that young adult e4+ (mean age 28) showed a pattern of hippocampal overrecruitment to novel stimuli when presented among well‐learned “familiar” stimuli. This was replicated in a follow‐up study in a slightly older age range (32–55), which also reported hippocampal overactivity during a Stroop task, where hippocampal activation was not to be expected (Trachtenberg, Filippini, Cheeseman, et al., 2012). Similarly, we have also reported hippocampal recruitment in e4+ (aged 18–28) during a covert attention task which does not usually elicit such activity (Rusted et al., 2013). It has been argued that such neural overrecruitment, seemingly evident across the lifespan in e4+ and possibly compensatory in nature, could drive cognitive performance advantages in young adulthood (Tuminello & Han, 2011). Some studies have reported that young adult e4+ can manifest cognitive advantages in certain domains, with e4+ outperforming e4− on measures of verbal fluency and prospective memory (Marchant, King, Tabet, & Rusted, 2010), and sustained and covert attention (Rusted et al., 2013), but larger studies using more general cognitive test batteries report no evidence for advantages (Bunce, Anstey, Burns, Christensen, & Easteal, 2011). Further work is required to resolve this issue, and interpret the significance of hippocampal overactivity in young adult e4+. Some MRI studies in young adult e4+ point to reduced volume in medial temporal lobe (MTL) (O'Dwyer et al., 2012; Wishart et al., 2006), and resting state studies have shown enhanced coactivation within hippocampal (Trachtenberg, Filippini, Ebmeier, et al., 2012) and default mode (Filippini et al., 2009; Su et al., 2015) networks, supporting a compensatory recruitment hypothesis.

Not all data are consistent with this, however. Mondadori et al., using an associative learning task, found that e4+ aged 20–25 actually showed diminishing hippocampal recruitment as the task progressed and this was linked to better performance. In contrast, e4− showed activity increases, leading the authors to suggest that e4+ might actually underrecruit neural resources under certain circumstances (Mondadori et al., 2007) and thus be more efficient in terms of neural recruitment. In young adulthood, therefore, a straightforward compensatory model might be overly simplistic.

In this study, we reverted to a classic subsequent memory paradigm, and extending the work outlined above, imaged both the acquisition and recognition phases so as to fully characterize hippocampal activation patterns in young adult e4+ during the task. Pupillometry data were acquired during the acquisition phase as an index of cognitive effort. Since compensatory neural recruitment likely reflects increased cognitive effort in older e4+ (Bondi et al., 2005) measuring cognitive effort could provide insight into whether differences in neural recruitment serve a similar compensatory role in younger e4+. Word stimuli were employed, to minimize luminance changes and eye movements. Evidence that pupil diameter can serve as an index of cognitive effort has been demonstrated across a variety of cognitive domains: for example, pupil size increases with task complexity during sentence comprehension (Just & Carpenter, 1993), and pitch discrimination (Schlemmer, Kulke, Kuchinke, & Van Der Meer, 2005). Pupil diameter has been shown to correlate with neural activity in dorsal attentional networks during a divided attention task (Alnaes et al., 2014), suggesting that pupil diameter could indicate the level of cognitive resources being directed towards a stimulus. In subsequent memory tasks, pupil diameter is enlarged to words that are subsequently remembered, versus forgotten (Papesh, Goldinger, & Hout, 2012). If neural recruitment differences reflect enhanced cognitive effort being deployed in e4+ as a means of achieving the same level of cognitive performance as in e4−, this should be detectable in the pupillometry measures. As such, we predicted genotype‐specific effects in pupil diameter (specifically, greater pupil diameter in e4+), and these effects were tested in two ways. First, we examined average pupil diameter in each condition (remembered/forgotten), by genotype. We then included pupil diameter as a covariate in the fMRI analyses to link pupillometry and neural activity measures. We did not anticipate any genotype differences in memory performance: a recent study using a word‐based subsequent memory task found that APOE status did not affect performance in young adults (Stening et al., 2016), as did the majority of studies using pictorial stimuli (outlined above), although it should be noted that these imaging studies have typically employed relatively small numbers and therefore might not have sufficient power to detect subtle memory impairment.

In terms of neural activation patterns, we predicted genotype‐specific differences in hippocampal activation, and a small volume correction was employed using a mask that incorporated both hippocampal and parahippocampal regions, bilaterally. This was used to determine whether levels of hippocampal activity showed any interactions between genotype and task condition, and specifically to test whether e4+ show greater hippocampal activity to trials that are subsequently remembered relative to those subsequently forgotten (as demonstrated by Dennis et al. (2009)).

## 2 Materials and Methods

### 2.1 Participants

Three hundred and twenty‐eight healthy participants (aged 18–28 years) were recruited from the University of Sussex. Protocols specified by the Human Tissue Act were followed throughout, participants consented to not being informed of their genotyping result, and volunteer call‐back was performed by a third party so that the researcher remained blind. *APOE* genotype was determined by buccal swab. Genotype analyses were performed by a third party (LGC Genomics, Hoddesdon, UK) using fluorescence‐based competitive allele‐specific polymerase chain reaction (KASPar) targeting two APOE single‐nucleotide polymorphisms (SNPs): rs429358 and rs7412. Invitation to the study was based on a random sampling so genotype status could not be inferred from an invitation to take part. Of these 328, 61 volunteers carried at least one e2 allele and were excluded. Sixty‐nine volunteers carried at least one e4 allele: 40 of these individuals were randomly invited to the study, of which 28 consented to take part. One hundred and ninety‐seven volunteers were homozygous e3 carriers and of these 50 were also randomly invited to the study, of which 26 consented to take part. Among the e4+ group, six participants were homozygous e4 carriers. Inclusion criteria were as follows: age 18–28, right handed, and fluent English speaker. Participants were excluded if they reported having high blood pressure, current treatment for a psychiatric condition, or failed the MRI safety screening.

The two groups were matched in age, but there was a trend towards an unequal gender balance, with more females than males overall (one‐tailed proportion test, z = 1.631, *p *= .052). For participants included in the fMRI analyses (whose recognition performance exceeded 50%), there was no significant difference in gender balance (one‐tailed proportion test, z = 0.316, *p* = .376), see Table 1. Nevertheless, gender was entered as a covariate in the behavioral, imaging, and pupillometry analyses.

**Table 1:** Volunteer characteristics for all participants, and those included in the fMRI analyses (recognition performance >50%)

All participants			Participants included in fMRI analyses		
Group	Age (years)	Gender	Group	Age (years)	Gender
e4− (*n *= 26)	20.91 ± 1.90	14F/12M	e4− (*n *= 19)	20.93 ± 2.14	8F/11M
e4+ (*n *= 28)	20.92 ± 2.59	19F/9M	e4+ (*n *= 21)	21.04 ± 2.77	13F/8M
t‐statistic	0.444, ns		t‐statistic	0.481, ns	

### 2.2 Experimental design

All participants volunteered under a written informed consent procedure approved by the Sussex University Schools of Psychology and Life Sciences Research Ethics Committee. Experimental procedures complied with the Code of Ethics of the World Medical Association (Declaration of Helsinki). The task was run as a component of a one‐hour scanner session. The acquisition phase of the task was presented as a semantic categorization task, and consisted of 100 words (all of which were 6 letters long) presented sequentially. Each word was presented at a central point on‐screen for 1 s. There was a variable ISI of 2.5–4.5 s. A mask (######) was presented between each stimulus. Participants were simply instructed to make a button press response to any word that described a profession, of which there were 8, quasirandomly distributed throughout the set, such that there were two profession words in each quarter. The acquisition phase duration was approximately 7.5 min. The surprise recognition phase began approximately 40 min after the acquisition phase. In the intervening period, participants completed some structural imaging and a vigilance task in the scanner (outcomes reported elsewhere). In the recognition phase, 180 words (the 100 words seen previously, plus 80 new words) were presented in random order using the same timings as in the acquisition phase. This time, participants were instructed to respond to each word, to indicate whether they thought it was previously studied in the acquisition (categorization task) phase (“old”) or a novel word (“new”). The recognition phase lasted approximately 13.5 min. The words used in both the acquisition and recognition phases were drawn from the MRC psycholinguistic database (RRID:SCR_014646) (http://www.psych.rl.ac.uk/MRC_Psych_Db.html) and matched for lexico‐semantic features of length (all words employed were 6 letters long), frequency, familiarity, and imageability, according to Kucera‐Francis norms, as this can impact recognition performance (Bauer, Olheiser, Altarriba, & Landi, 2009).

### 2.3 fMRI recording and analysis

fMRI datasets sensitive to BOLD (blood oxygen level dependent) contrast were acquired at 1.5 T (Siemens Avanto). To minimize signal artifacts originating from the sinuses, axial slices were tilted 30° from intercommissural plane. Thirty‐six 3 mm slices (0.75 mm interslice gap) were acquired with an in‐plane resolution of 3 mm × 3 mm (TR = 3300 ms per volume, TE = 50 ms). Images were preprocessed using SPM8 (RRID:SCR_007037) (http://www.fil.ion.ucl.ac.uk/spm/). Raw T2 volumes were spatially realigned and unwarped, spatially normalized to standard space and smoothed (8 mm kernel). fMRI data were analyzed with the standard hierarchal model approach employed in SPM. Design matrices were constructed for each participant's acquisition phase, which modeled subsequently remembered, subsequently forgotten, and profession sort trials as separate regressors. Design matrices were also constructed for each recognition phase, which modeled profession sort, “Old” correct, “Old” incorrect, “New” correct and “New” incorrect trials as separate regressors. Movement parameters were also entered. Processing of fMRI data was performed blind to group membership. For the acquisition phase, contrasts for subsequently remembered and forgotten trials were entered at the first level, and effects of condition (remembered/forgotten) and genotype (e4−/e4+) were analyzed at the second level in a full factorial design. For the recognition phase, contrasts for “Old” correct, “Old” incorrect, “New” correct and “New” incorrect were entered at the first level. At the second level, effects of condition for correct responses (i.e., “New” correct/ “Old” correct) and genotype (e4−/e4+) were analyzed using a flexible factorial to test for the effects of condition and condition by genotype interaction, followed by a two sample t‐test (with the 2 conditions averaged) to test for main effects of genotype. In addition, a separate model examined effect of condition when participants made a “new” judgment (i.e., “New” correct, “Old” incorrect). Again, a flexible factorial followed by t‐test was employed. We thank an anonymous reviewer for suggesting this procedure (the original approach was to utilize SPM's full factorial design, but for mixed within‐ and between‐subject analyses this can be problematic as only one error term is used). We thank the same reviewer for suggesting the multiple regression analysis (p.8).

Recognition performance (proportion of studied words correctly identified) and gender were entered as covariates. The recognition performance covariate was entered to control for between‐subject variance in performance; furthermore, to ensure we were only analyzing data from participants who performed the task correctly (and ensure sufficient trials in the subsequently remembered condition), we excluded participants whose percentage of subsequently remembered words was <50%.

**Figure 1:**
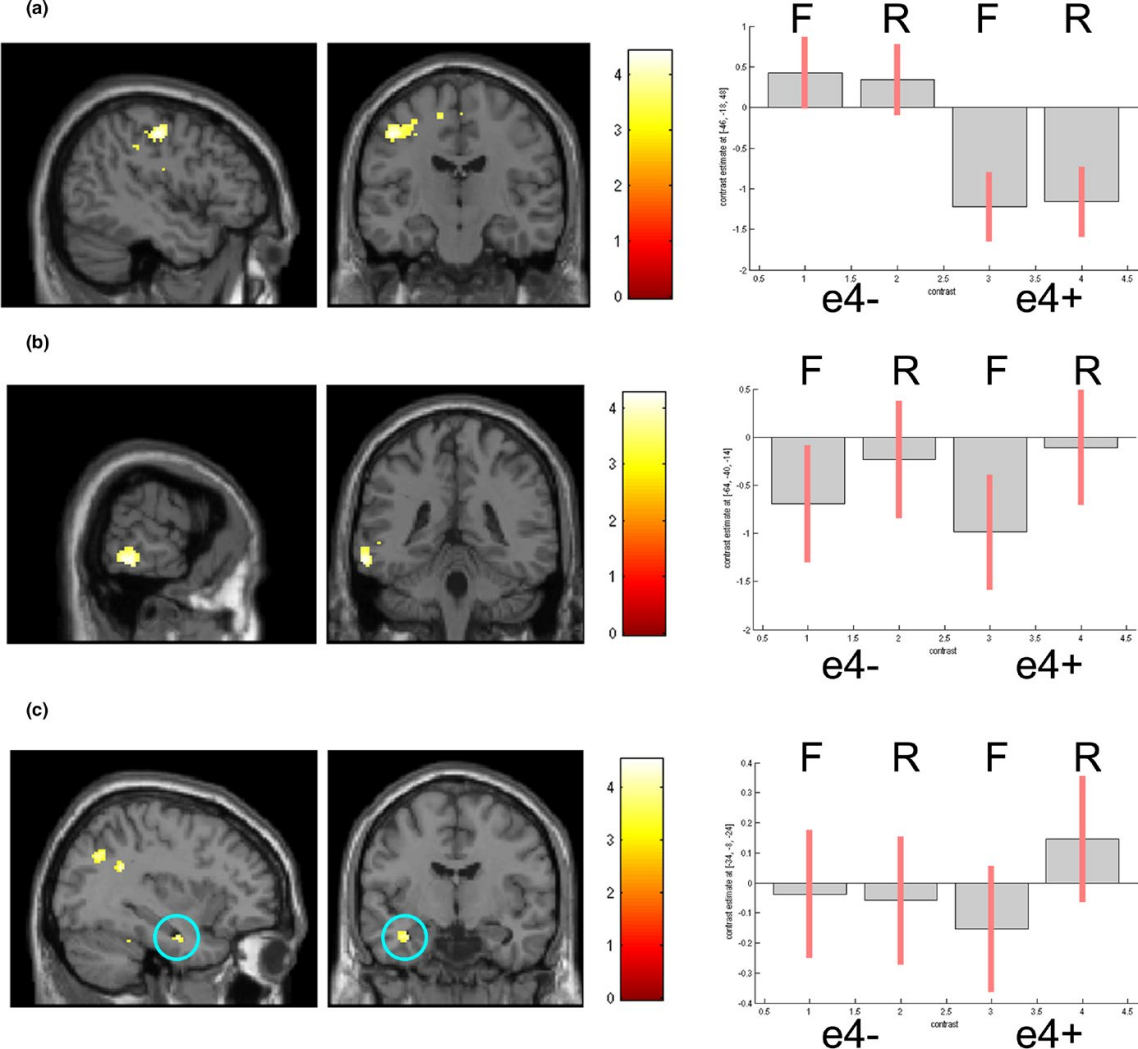
Activation maps (at *p* < .001 unc) and associated parameter estimates with 90% CI (F = Forgotten, R = Remembered) showing (a) Greater overall activity in left BA4/BA6 in e4− (b) Activity in left middle temporal lobe differentiates remembered and forgotten trials (c) Only e4+ show greater activity in left hippocampus to remembered trials

**Figure 2:**
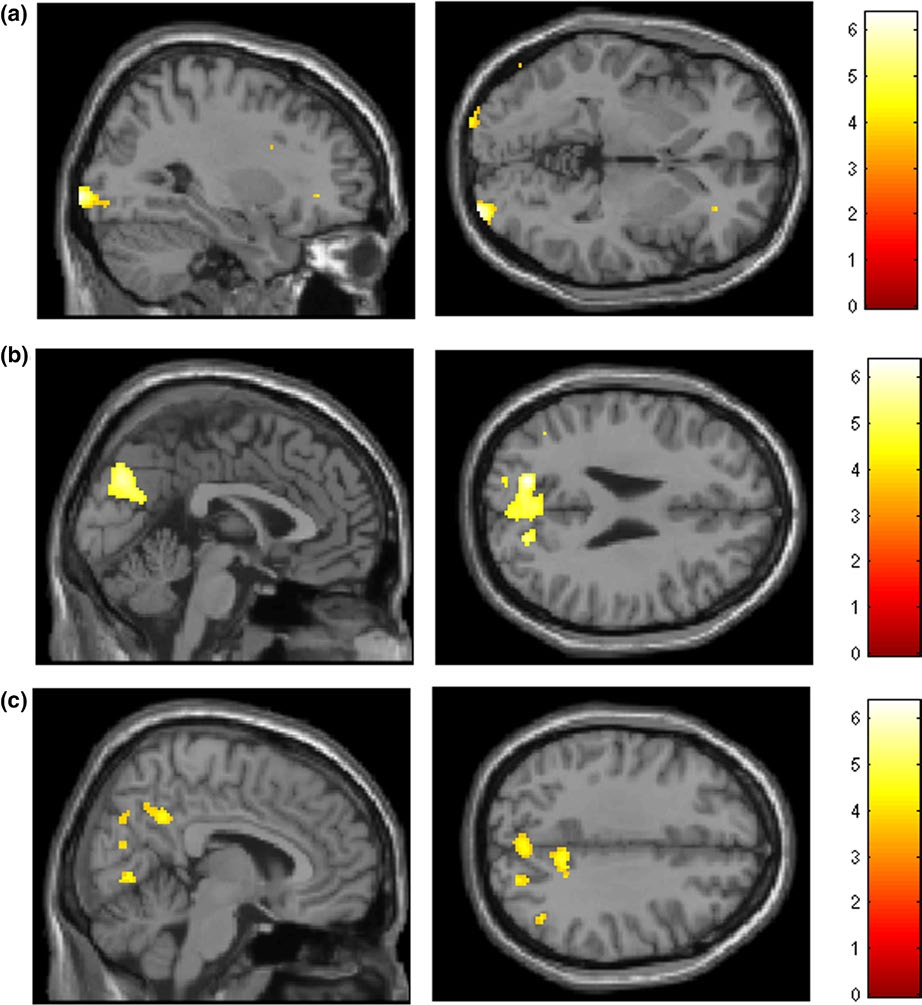
Activation maps (at *p* < .001 unc.) showing variance explained by pupil diameter as a 2nd‐level covariate in (a) BA18 (b) anterior cuneus/SPL and (c) precuneus

**Figure 3:**
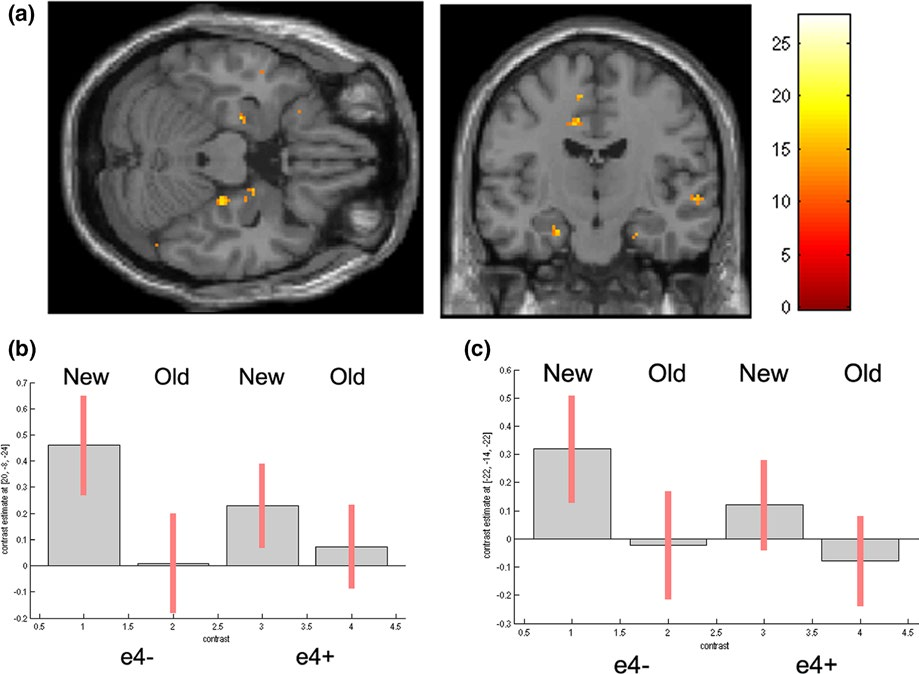
(a) Bilateral hippocampal activity to “New”> “Old” contrast in recognition phase. Parameter estimates and 90% C.I. for cluster in (b) right hippocampus (c) left hippocampus

The small volume correction for the MTL was performed using a mask generated by the Wake Forest University PickAtlas (RRID:SCR_007378) (Maldjian, Laurienti, Kraft, & Burdette, 2003), incorporating hippocampal and parahippocampal regions. The significance threshold was set at *p* < .05 FWE‐corrected (cluster level). When the small volume correction was applied, the significance threshold was set at *p* < .05 FWE‐corrected (peak level). Images (Figures 1, 2 and 3) were thresholded at *p* < .001 uncorrected. Parameter estimates and 90% confidence intervals (Figures 1 and 3b,c) were extracted using the corresponding coordinates from Tables 4 and 7, respectively.

### 2.4 Pupillometry recording and analysis

Pupil diameter was recorded throughout the fMRI acquisition using an ASL Eyetrac 6 system with a 120 Hz sampling rate. Data were converted using ASL's EyeNal software package (RRID:SCR_005997). Data were quality checked and deemed usable for 40 participants (20 e4+ and 20 e4−). The criteria for including a participant was that >75% of data samples had to be available for all word stimuli. Intermittent tracking of the pupil, resulting in insufficient data samples, was due to use of the MRI‐safe goggles, light‐colored irises, or head position in the coil. For each participant, average pupil diameter was calculated for each word (incorporating the time period when the word was on‐screen, and the mask that followed it), averages were then calculated for words subsequently remembered/forgotten. Data were analyzed using a within‐subjects ANOVA, with gender as a covariate. Furthermore, to investigate the neural correlates of pupil diameter, average pupil diameter for each participant was added as a covariate to the full factorial model for the acquisition phase (described above). For each participant, two values were entered: average pupil diameter to remembered trials and average pupil diameter to forgotten trials. These values were entered against each participant's corresponding first‐level contrast image. The effect of this covariate was then examined using a second‐level contrast, allowing us to determine where neural activity during forgotten and remembered trials correlated with pupil diameter in each participant.

## 3 Results

### 3.1 Behavioral data

#### 3.1.1 Acquisition phase

Participants were accurate in identifying the eight profession words (see Tables 2 and 3). The number of false alarms was low: Mean = 0.87, sd = 1.12. There were no effects of genotype.

**Table 2:** Proportion correct and s.d. for Sort trials at acquisition (*n *= 8), “Old” words presented at recognition (*n *= 92), “New” words at recognition (*n *= 80). Data presented for all participants, and the group included in the fMRI analyses, whose recognition performance exceeded 50%. There were no genotype effects

Group	Sort	“Old”	“New”
All (*n *= 54)	0.95 ± 0.07	0.56 ± 0.14	0.78 ± 0.12
Acc>0.5 (*n *= 40)	0.96 ± 0.03	0.61 ± 0.10	0.75 ± 0.12

**Table 3:** Proportion correct and s.d. for “Old” and “New” words presented at recognition, and the discriminability index d’, for the group included in the fMRI analyses (by genotype)

	“Old”	“New”	d’
e4− (*n *= 19)	0.60 ± 0.11	0.72 ± 0.14	0.90 ± 0.43
e4+ (*n *= 21)	0.57 ± 0.10	0.77 ± 0.10	0.97 ± 0.38

#### 3.1.2 Recognition phase

See Tables 2 and 3. There were no effects of genotype or interactions with stimulus type (“Old”/ “New”). Performance was poor, however, with a number of participants failing to recognize over half of the words presented in the acquisition phase. Participants who failed to identify at least 50% of studied words in the recognition phase were excluded from the fMRI analysis. This criterion meant that 7 e4− and 7 e4+ were excluded (Table 2). Therefore fMRI datasets from 19 e4− and 21 e4+ were analyzed, recognition performance in this group (including the discriminability index d’) is shown in Table 3. To explore the recognition performance patterns further, we investigated whether position in the word list at acquisition had an effect on likelihood of recognition. Evidence for a primacy effect was found: words presented earlier in the list were significantly more likely to be successfully classified as “Old” in the recognition phase.

### 3.2 Neuroimaging data

#### 3.2.1 Acquisition phase

##### Effects of genotype

The contrast e4+>e4− over both conditions (remembered/forgotten) showed no genotype effects. The contrast e4−>e4+ revealed effects in left BA6 (Table 4, Figure 1a).

**Table 4:** Acquisition phase: fMRI results by contrast

Contrast	Region	Vox	x, y, z	*p* value
e4−>e4+	Left BA4/BA6	353	−46, −17, 48	*p* < .001
Remembered>Forgotten (all subjects)	Left middle temporal	172	−62, −40, −14	*p* = .020
Remembered>Forgotten (e4+)	Left hippocampus	11	−34, −8, −24	*p* = .045 after S.V.C.
Effect of pupil diameter as 2nd‐level covariate	Right BA18	120	32, −88, −6	*p* < .001
	Left anterior cuneus	775	−14, −74, 28	*p* = .035
	Right precuneus	129	10, −52, 36	*p* = .026

##### Remembered>Forgotten

Across all subjects, significantly greater activation was seen in a left middle temporal region to subsequently remembered over forgotten trials (Table 4, Figure 1b).

##### Interaction with genotype

No significant interaction was observed between condition (Remembered/Forgotten) and genotype.

##### Remembered>Forgotten in e4+

In accordance with our specific predictions, we examined activity related to Remembered>Forgotten in e4+ using a SVC incorporating bilateral parahippocampus and hippocampus. Activity was observed in left hippocampus (Table 4, Figure 1c). A similar contrast in e4− showed no such activity.

##### Pupillometry data

Average pupil diameter during acquisition for subsequently remembered and forgotten words is shown in Table 5. Data met all assumptions for use of parametric tests. Analyzed using ANOVA, there was a main (within‐subjects) effect of condition, with significantly greater pupil diameter for subsequently remembered words (*F *= 13.611, *p* = .001). There was no main effect of genotype (*F *= 0.003, *p* = .953) and no genotype by condition interaction (*F *= 1.623, *p* = .210).

**Table 5:** Acquisition phase: Average pupil diameter to subsequently remembered and forgotten words; F and *p* values (two‐tailed) from a repeated measures ANOVA testing for a within‐subjects main effect of condition (remembered/forgotten)

	Mean (arbitrary units)	*SD*	*F*	*p* value
All subjects				
Remembered	36.63	8.62	13.611	.001
Forgotten	35.72	9.23		
e4−				
Remembered	36.72	8.64	12.913	.002
Forgotten	35.44	9.47		
e4+				
Remembered	36.51	8.82	2.794	.111
Forgotten	35.90	9.21		

##### Adding pupil diameter as 2nd‐level covariate

To investigate the neural correlates of pupil diameter, pupil diameter was added as a covariate to the 2nd‐level model. Two values were entered per participant, corresponding to the average over remembered and forgotten trials. This covariate was seen to explain variance in a posterior midline region (anterior cuneus extending into superior parietal regions), extrastriate visual cortex (BA18) and precuneus (Table 6, Figure 2a,b,c). Beta estimates for each participant by condition (forgotten/remembered) were extracted for the peak voxel in each cluster (i.e., 2 values were extracted per participant, corresponding to mean over forgotten and mean over remembered). To test for genotype effects, these were correlated against mean pupil diameter by condition for each participant. As 6 correlations were assessed, a Bonferroni‐adjusted significance threshold of *p* < .00833 was employed. In anterior cuneus, betas showed a negative correlation with pupil diameter for both e4− and e4+. In BA18 a significant positive correlation was seen in e4− only, whereas in precuneus significant negative correlation was seen in e4− only (Table 6). Plots of pupil diameter against beta estimates are included in the supplementary materials, for each of these regions. Multiple regression confirmed a main effect of genotype on the interaction with pupil diameter, in BA18 (78 voxels, 30, −84, −5, *p* = .039 FWE – corrected) and in precuneus (83 voxels, 8, −50, 34, *p* = .042 FWE – corrected).

**Table 6:** Correlations between peak voxel beta values and mean pupil diameter, by genotype group

Region	Coordinates (x, y, z)	Pearson's r (*p* value)	
		e4−	e4+
BA18	32, −88, −6	*r *= .684 (*p* < .001)	*r *= −.009 (*p* = .961)
Anterior cuneus/SPL	−14, −74, 28	*r *= −.739 (*p* < .001)	*r *= −.493 (*p* = .003)
Precuneus	10, −52, 36	*r *= −.731 (*p* < .001)	*r *= .148 (*p* = .403)

As these correlations indicated genotype‐specific effects, we then conducted ANOVA on the pupil diameter data, separately for each genotype group. A main (within‐subject) effect of condition was significant only in e4− (*F *= 12.91, *p* = .002, Table 5).

#### 3.2.2 Recognition phase

##### Correctly identified “Old” > Correctly identified “New” words

Significant effects were seen in bilateral insula, left inferior parietal, and left orbitofrontal (see Table 7). There were no main effects of genotype group.

**Table 7:** Recognition phase: fMRI results by contrast

Contrast	Region	Vox	X, y, z	*p* value (FWE‐corrected)
Old>New	Left insula	448	−32, 25, −6	*p* < .001
	Right insula	251	34, −26, −8	*p* = .001
	Left inf parietal	430	−32, −52, 32	*p* < .001
	Left orbitofrontal	431	−22, 64, −2	*p* = .005
	ACC/middle cingulate	368	−4, 35, 34	*p* < .001
New>Old	Left BA18	401	−20, −86, 21	*p* < .001
	Right BA18	255	20, −87, 26	*p* = .005
	Right Hippocampus	47	20, −9, −24	*p* = .009
	Left Hippocampus	12	−22, −14, −22	*p* = .044 after bilateral S.V.C.
Interaction with genotype	Right Hippocampus	6	20, −8, −25	*p* = .048 after bilateral S.V.C.

##### Correctly identified “New” > Correctly identified “Old” words

Significant effects were seen in bilateral BA18 and bilateral hippocampus (see Table 7, Figure 3). There were no main effects of genotype group.

##### Interaction with genotype

A significant interaction between condition (Correctly identified “New”/ Correctly identified “Old”) and genotype was seen in right hippocampus (see Table 7, Figure 3b). A follow‐up t‐test (“New”> “Old”) was significant in e4− (Right hippocampus, 38 vox, *p* = .043 FWE‐corrected, cluster level) but not in e4+ (3 vox, *p* = .241 FWE‐corrected, cluster level).

#### 3.2.3 Recognition phase – “New” responses

In a separate 2nd‐level model, we investigated effect of condition (“New”/ “Old”) when participants responded “New” (i.e., contrasting correctly identified “New” with incorrectly identified “Old”). There was no effect of condition and no interaction with genotype.

## 4 Discussion

In this study, we set out to explore *APOE* effects on subsequent memory performance in young adults, specifically with reference to previous findings suggesting a pattern of hippocampal overactivity in e4+. In line with previous studies using subsequent memory paradigms (Dennis et al., 2009; Filippini et al., 2009), we found no genotype differences on recognition performance. Participants returned near‐perfect scores on the sorting of profession words during the acquisition phase, indicating that they paid attention to the word stimuli. Recognition performance in the retrieval phase was necessarily reduced by the use of word, as opposed to picture, stimuli, by the employment of an incidental memory procedure, and by the 40‐minute filled delay between acquisition and recognition phases. Although recognition rates were low, they followed the anticipated pattern: serial position effects were evident, with words presented nearer the beginning of the acquisition phase more likely to be recognized when represented forty minutes later.

For the neuroimaging data analyses, we contrasted activity to subsequently remembered against subsequently forgotten words in the acquisition phase. In the recognition phase only correct responses were considered, contrasting “Old” against “New” words. To ensure reliable data, we excluded participants from the neuroimaging analyses if they failed to identify at least 50% of previously studied words in the recognition phase. This meant that seven participants from each genotype group were excluded. The poor levels of performance necessitating such exclusions should be noted as a shortcoming of this study.

At acquisition, e4+ showed less activity in BA4/BA6 relative to e4−, across both subsequently remembered and forgotten words. We have previously demonstrated genotype effects in BA6 on a covert attention task (Rusted et al., 2013), in which young adult e4+ were faster at attentional switching. In that study, e4+ showed greater activity in BA6 and precuneus, which previous studies have linked to better performance on sustained attention tasks (Lawrence, Ross, Hoffmann, Garavan, & Stein, 2003); indeed, we also found young adult e4+ to show enhanced sustained attention performance (Rusted et al., 2013). Here, e4+ were seen to consistently underactivate BA4/BA6. The attentional demands of the acquisition task used here are likely considerably less than those of the covert attention task employed previously, suggesting that activity in this region in e4+ might be more labile and sensitive to task demand than in e4−. APOE effects in BA6 have been identified at mid‐age, with e4+ showing diminished left BA6 recruitment during an object‐naming task, alongside decreased activity in occipital and medial temporal lobes (Tomaszewki Farias, Harrington, Broomand, & Seyal, 2005).

On the basis of previous findings (Dennis et al., 2009), we expected e4+ to show greater hippocampal activity to subsequently remembered words at acquisition, compared to e4−. Consistent with this, activity in left hippocampus was seen to differentiate remembered and forgotten words in e4+ only. This demonstrates that at acquisition hippocampal overactivation in e4+ is detectable using a standard word‐based subsequent memory paradigm; previous studies have employed pictorial stimuli, which are more likely to elicit hippocampal recruitment. Indeed, a study looking at effect of stimulus type has shown that although remembered picture stimuli activated bilateral MTL, activation to word stimuli did not reach significance in MTL at all (Kirchhoff, Wagner, Maril, & Stern, 2000). Dennis et al. (2009) found bilateral hippocampal effects in e4+, consistent with the use of picture stimuli (picture stimuli engage both hemispheres, whereas word encoding is left lateralized (Kelley et al., 1998)).

In the recognition phase, correctly identified “New” and “Old” words were contrasted and in line with previous studies (Filippini et al., 2009), greater activity to “New” words was seen in MTL regions, with differential activity also present in insula, cingulate, inferior parietal, and early visual regions (Filippini et al., 2009; Golby et al., 2005). Novel stimuli elicited activity in right hippocampus, with activity in left hippocampus occurring at the trend level. Furthermore, a genotype by condition (Old/New) interaction was present in the right hippocampus. Follow‐up tests showed that the hippocampal New>Old effect was significant in e4−, but not e4+. This contrasts with the findings of Filippini et al. (2009) who reported greater activity to novel words in young adult e4+. However, their paradigm differed from ours in that participants were repeatedly familiarized with the “old” stimuli. Work in healthy older e4+ (aged 58–65) similarly reported hippocampal overactivation in e4+ in a novelty paradigm (Fleisher et al., 2005), whereas hippocampal activity in early‐stage AD patients tends to not differentiate novel and familiar words (Golby et al., 2005). It is not clear why the young adult e4+ under test here showed enhanced activity at acquisition specific to subsequently remembered items (while e4− did not), followed by a hippocampal underactivation to novel items at recognition. Clearly these results indicate that e4+ do not simply show a consistent pattern of hippocampal overactivity. Supporting evidence can be drawn from work by Mondadori et al. showing decreases in hippocampal activity across learning runs in an associative memory task, in young adult e4+ (Mondadori et al., 2007). Interestingly, a study in healthy older e4+ (mean age 60) showed a similar pattern of findings. e4+ showed increased activity at acquisition to subsequently remembered items in prefrontal, temporal, and parietal regions, whereas successful recognition was linked to lower activity in amygdala and prefrontal regions (Kukolja et al., 2010). Since these older e4+ showed worse performance, this was interpreted as being indicative of premature neural decline. Although the study population was some four decades older than the one employed here, the authors reached the same conclusion, namely that the direction of e4+ effects on neural activity varies according to task phase.

A novel aspect of the current work was the inclusion of pupillometry measures. Pupil diameter indexes cognitive processing as well as general arousal state, and we collected pupil diameter throughout the acquisition phase. It has been suggested that the neural overactivation frequently observed in e4+ might be compensatory in nature and reflect greater deployment of cognitive effort (Bondi et al., 2005): we thus predicted genotype‐specific effects in pupil diameter. Previous studies point to a reliable remembered/forgotten effect, where pupil diameter is greater for words that are subsequently remembered: this is thought to reflect the higher level of cognitive effort engaged to words that are subsequently remembered (Papesh et al., 2012). Our pupillometry results showed this remembered/forgotten effect, but in e4− only. Although there was no condition by genotype interaction, genotype‐specific analyses showed that in e4+, there was no relationship between pupil diameter and whether a word was subsequently remembered or forgotten: allocation of cognitive effort to a stimulus did not predict whether it was subsequently remembered. When pupil diameter was introduced as a covariate in the fMRI analyses, it was seen to explain variance across three separate clusters in occipital lobe and precuneus, but effects were genotype‐specific. Activity in extrastriate regions showed a positive relationship with pupil diameter, but only in e4−. This suggests that, in this group, greater pupil diameter is linked to enhanced processing of the word stimulus and a higher likelihood that it is subsequently remembered. A posterior midline region (encompassing posterior cuneus and superior parietal regions) showed a negative relationship across all participants. In addition, we found that activity in precuneus showed a negative relationship in e4− only. This is consistent with previous work linking DMN downregulation to subsequent memory success. The precuneus and posterior cingulate cortex form a core node of the DMN; DMN downregulation might signal a shift in attention from internal processes to external stimuli, thus increasing the likelihood of subsequent recall (Anticevic, Repovs, Shulman, & Barch, 2010; Daselaar, Prince, & Cabeza, 2004; Otten & Rugg, 2001). Greater coactivation within the DMN has been previously demonstrated in young adult e4+ during the resting state (Filippini et al., 2009; Sheline et al., 2010). These coactivation differences might mean that DMN shows less deactivation when attention is directed to external stimuli in e4+, which could underlie the pupillometry effects found here. Interestingly, Lustig et al. (2003) used an incidental encoding task to show that, whereas young adults showed precuneus deactivation to remembered items, healthy older adults did not. Here, precuneus activity did not covary with pupil diameter in e4+, suggesting a lack of responsivity similar to that seen in older adults, a pattern we have identified previously in mid‐age e4+ (Evans et al., 2014). However it should be noted that, since the fMRI data showed no overall main effects of genotype within the DMN, this interpretation requires further exploration.

In conclusion, we have shown that previous findings of hippocampal overactivity in young adult e4+ to subsequently remembered items generalize to a standard word‐based paradigm. Typically, hippocampal activity in the acquisition phase to subsequently remembered items is shown when the paradigm includes tests of source memory or associative memory, rather than straightforward recognition judgments, suggesting that hippocampus underlies recollection, rather than familiarity‐based decisions (Shrager, Kirwan, & Squire, 2008). Consequently, hippocampal activation to remembered items depends on the nature of the incidental task: when the task promotes the formation of rich episodic memories, hippocampal activation is evident (de Chastelaine & Rugg, 2015). Given that e4+ showed hippocampal activity to remembered stimuli, whereas e4− did not, this suggests that e4+ require hippocampal recruitment during incidental encoding if items are to be successfully recovered at recognition. This overrecruitment occurred in the context of genotype‐specific effects in the pupillometry data, with links between pupil diameter, neural activity, and cognitive performance disrupted in e4+. This could be due to coactivation differences within DMN reported elsewhere. These findings (that hippocampal recruitment, rather than the deployment of cognitive effort, differentiates remembered from forgotten words in e4+) need to be explored further. Since hippocampal overactivation did not map onto pupillometry measures, it seems that if this overactivity is compensatory, it involves a mechanism not linked to cognitive effort. Indeed, deployment of cognitive effort did not link to subsequent memory performance in e4+. Interestingly, e4+ showed the opposite pattern in the recognition phase, with hippocampal activity now failing to differentiate “new” and “old” items. In contrast, e4− showed the normal novelty effect with hippocampus activating to novel stimuli. Although this also needs to be replicated, it does suggest that an account that posits consistent hippocampal overrecruitment in e4+ might be overly simple: while studies have reported that e4+ may recruit the hippocampus even when it is not appropriate to task demands (Rusted et al., 2013; Trachtenberg, Filippini, Cheeseman, et al., 2012), here e4+ failed to recruit hippocampus when it was task relevant, suggesting that hippocampal recruitment in e4+ is inconsistent, certainly abnormal, and is not always in the direction of overactivity. More work is needed to elucidate the relationship between e4 genotype, neural activity patterns and cognitive performance, but this study provides further evidence that, in young adulthood, *APOE* genotype influences brain activation patterns even when behavioral performance differences are absent.

## Conflicts of Interest

The authors have no conflicts of interest to declare.
