# Supplementary material for: Disrupted neural activity patterns to novelty and effort in young adult APOE‐e4 carriers performing a subsequent memory task
Source: Brain Behav. 2017 Jan 5;7(2):e00612. doi: 10.1002/brb3.612 (PMC5318365; doi:10.1002/brb3.612)
Supplement: Supplementary file 1 [file BRB3-7-e00612-s001.docx]

**Supplementary materials**


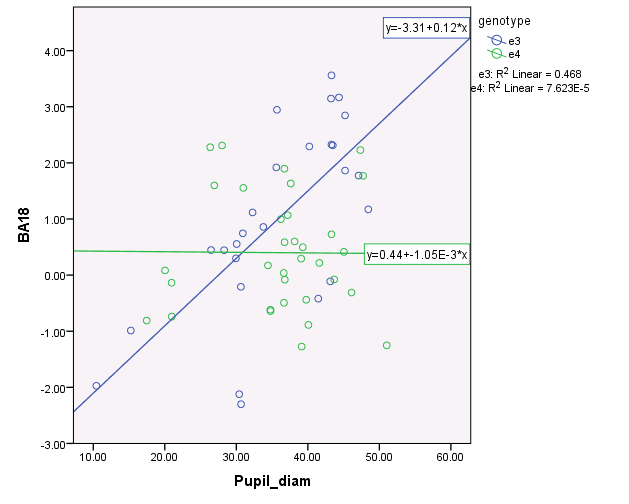


**Figure 1.** Acquisition phase: Pupil diameter plotted against beta estimates from BA18 (32, -88, -6), for each participant (2 data points for each participant, corresponding to mean over forgotten and remembered trials). Regression line fitted per genotype group.


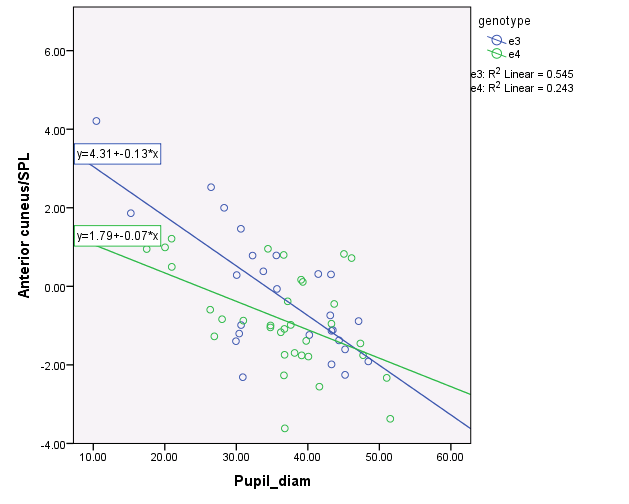


**Figure 2.** Acquisition phase: Pupil diameter plotted against beta estimates from Anterior cuneus/SPL (-14, -74, 28), for each participant (2 data points for each participant, corresponding to mean over forgotten and remembered trials). Regression line fitted per genotype group.


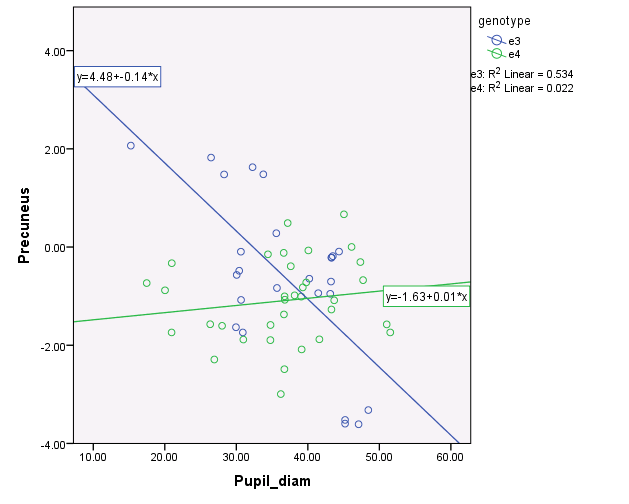


**Figure 3.** Acquisition phase: Pupil diameter plotted against beta estimates from Precuneus (10, -52, 36), for each participant (2 data points for each participant, corresponding to mean over forgotten and remembered trials). Regression line fitted per genotype group.
